# Canine Platelet-Rich Plasma Systems: A Prospective Analysis

**DOI:** 10.3389/fvets.2015.00073

**Published:** 2016-01-05

**Authors:** Brittany Jean Carr, Sherman O. Canapp, David R. Mason, Catherine Cox, Theresa Hess

**Affiliations:** ^1^Veterinary Orthopedic and Sports Medicine, Annapolis Junction, MD, USA; ^2^Las Vegas Veterinary Specialty Center, Las Vegas, NV, USA

**Keywords:** platelet-rich plasma, autologous conditioned plasma, leukocyte-rich platelet-rich plasma, leukocyte-poor platelet-rich plasma, platelet count

## Abstract

**Objective:**

To quantitate key parameters of the platelet-rich plasma (PRP) product from five commercial canine PRP systems in healthy, adult canines.

**Materials and Methods:**

A prospective study was performed from January 2013 to April 2014. Five commercial systems were analyzed using 10 healthy dogs per system.^1^^–^^5^ Blood was obtained according to the manufacturer’s protocol for each system. The mean baseline whole blood platelet, RBC, WBC, neutrophil, monocyte, and lymphocyte concentrations were determined for each PRP system. All blood samples were processed according to the manufacturer’s protocols. The mean PRP product platelet, RBC, WBC, neutrophil, monocyte, and lymphocyte concentrations were determined for each PRP system. These values were then compared to the mean baseline values. Comparisons of mean whole blood and mean PRP product parameters were calculated using a paired *t*-test with significance established at *p* = 0.05.^6^

**Results:**

Platelet concentration was significantly increased for System 1 (*p* = 0.0088) and System 3 (*p* < 0.0001), and was significantly decreased for System 2 (*p* < 0.0001). All five systems significantly decreased the red blood cell concentration (*p* < 0.0001 for each system comparison). Neutrophil concentration was significantly decreased for System 2, System 3, and System 4 (*p* < 0.0001 for each system comparison). Neutrophil concentration was significantly increased for System 5 (*p* = 0.0089).

**Clinical Relevance:**

The systems with the highest platelet yield were System 1 and System 3. System 3 increased platelet concentration while significantly reducing the RBC and neutrophil concentrations. Further study is indicated to assess the efficacy of PRP therapy in canines, the efficacy of canine PRP systems, and the clinical applications for PRP therapy in dogs.

## Introduction

Platelet-rich plasma (PRP) is an autogenous fluid concentrate composed primarily of platelets and growth factors. Recent studies have shown PRP to mediate healing by supplying growth factors, cytokines, chemokines, and other bioactive compounds (1–7). Initially, PRP’s first clinical applications were limited to dentistry and maxillofacial surgery to improve bone healing. However, PRP currently has much broader clinical applications, extending to orthopedic surgery and sports medicine. Recent studies have shown PRP to be efficacious in managing many different orthopedic conditions, including osteoarthritis and soft tissue injuries (3, 4, 7–31).

Platelets play roles in both hemostasis and wound healing. Platelets contain granules that release growth factors to stimulate other cells of the body to migrate to the area of trauma, thus facilitating tissue healing. It is the growth factors contained within the platelets that are of significance for tissue healing. These growth factors include platelet-derived growth factor (PDGF), transforming growth factor-β1 (TGF-β1), transforming growth factor-β2 (TGF-β2), vascular endothelial growth factor (VEGF), basic fibroblastic growth factor (bFGF), and epidermal growth factor (EGF) (1–4, 6). Many of the growth factors found in PRP have been shown to act either individually or synergistically to enhance cellular migration and proliferation, angiogenesis, and matrix deposition to promote tendon and wound healing, aid in bone healing, and counteract the cartilage breakdown that is associated with osteoarthritis (2–8, 10, 13, 19, 22, 26, 29, 31) Thus, PRP has been used to manage many different orthopedic conditions. A number of studies have supported the use of PRP for soft tissue healing (10, 11, 19–21, 24–27, 30). A recent double-blinded, randomized controlled trial showed that patients with patellar tendinopathy treated with PRP had greater function and less pain than patients in the control group (11). Multiple studies have also documented the use of PRP for management of osteoarthritis (7, 8, 12–18, 22, 23). One recent prospective, blinded, randomized trial showed a single dose of PRP to be more effective than a placebo for improving function in humans with knee osteoarthritis (22). Furthermore, recent studies have also shown that platelets recruit, stimulate, and provide a scaffold for stem cells, supporting its use with stem cells to stimulate healing (27, 31–39). Hence, PRP has also been used in conjunction with stem cell therapy to aid in cartilage, bone, and soft tissue healing.

Multiple formulations of PRP have been developed and studied. Previous studies in humans have reported that the ideal PRP product should have anywhere from a four- to sevenfold increase in platelets (2–4, 6). However, platelet concentration is not the only important component of a PRP product. Inclusion or exclusion of mononuclear cells, neutrophils, and red blood cells not only define an autologous platelet product but have also been reported to affect the clinical efficacy of the product and play major roles affecting the inflammatory responses after PRP injection (2, 5, 10, 19–22, 40–44). In general, it is believed that red blood cells and neutrophils should be reduced as they have an inflammatory effect, while the effect of mononuclear cells remains largely unknown (36, 40, 41, 45–48).

Multiple commercial PRP separation systems have been developed for both human and equine use. PRP products from different commercially available PRP separation systems often have variations in their concentrations of platelets, WBC, and growth factors (6, 43). Furthermore, while these systems may have been previously validated for human and/or equine use, there is limited research supporting their validation of a PRP system for canine use. In fact, a recent study revealed that PRP systems validated for human and/or equine use may not yield similar or consistent results in the canine (44). Another recent study evaluated some of the commercial canine PRP systems and also found inconsistent results; however, this study failed to perform statistical analysis and WBC differential on the PRP products (49). The purpose of this study was to compare key parameters of the PRP product from five of the most commonly used commercial canine PRP systems in healthy, adult canines.

## Materials and Methods

A prospective study involving two small animal surgery specialty centers was performed from January 2013 to April 2014. In accordance with AAALAC International Rules of Accreditation, this study was performed with the approval of the VOSM Research Committee and with owner consent. In this study, all dogs who participated were client-owned dogs deemed healthy by a veterinarian. All clients volunteered their dog for the study and provided written consent as required by Veterinary Orthopedic and Sports Medicine Group for every study participant. All dogs that participated in the study were directly overseen by a veterinarian to ensure no harm was incurred during study participation.

The following five commercial systems were prospectively analyzed and labeled as follows: System 1,^1^ System 2,^2^ System 3,^3^ System 4,^4^ and System 5.^5^ Data for Systems 1, 3, and 4 were collected at Center 1,^7^ while data for Systems 2 and 5 were collected at Center 2 (See footnote 6). Ten adult, healthy dogs with no known previous or current medical problems were used for each PRP system. Two of the canines were used for both System 3 and System 4. In this case, the blood samples were collected on separate days, 30 days apart from each other. One investigator was appointed at each center to perform blood collection and sample processing. The required volume of blood was then obtained from each dog according to the manufacturer’s protocol and specifications for each PRP system (Table 1). For System 1, System 2, System 3, and System 5, all blood samples were obtained from the jugular vein using an 18-gage-butterfly needle. All blood samples for System 4 were collected from the jugular vein; however, System 4 required the use of a 21-gage-butterfly needle vacutainer as blood was collected directly into the sodium citrate tubes provided by the manufacturer for processing. A baseline blood WBC differential, RBC concentration, and platelet concentration were obtained on all dogs using an in-house hematology analyzer that had been calibrated according to manufacturer standards.^8^ The mean baseline whole blood platelet, RBC, WBC, neutrophil, monocyte, and lymphocyte concentrations were determined for each PRP system. Baseline concentrations were obtained immediately following blood draw for all dogs. All blood samples were processed immediately following blood draw according to the protocols established by the manufacturer. PRP product concentrations were obtained immediately following processing. The platelet, RBC, WBC, neutrophil, monocyte, and lymphocyte concentrations were obtained for all processed samples. The mean PRP product platelet, RBC, WBC, neutrophil, monocyte, and lymphocyte concentrations were determined for each PRP system. The D’Agostino & Pearson Omnibus normality test was performed on the differences between PRP and Whole Blood for each data set with significance established at *p* = 0.01. All data sets were found to be normally distributed. Data were then analyzed using a paired *t*-test. These values were analyzed using statistical software.^9^ Significance was established at *p* = 0.05.

**Table 1:** **PRP system protocols summary**.

System	Centrifugation or filtration	Volume of blood required	Anticoagulant	Volume of anticoagulant	Size of syringe used	PRP product volume
1	Centrifugation	50 mL	ACD-A	10 mL	60 mL	10 mL
2	Centrifugation	16 mL	ACD-A	1.5 mL	20 mL	4–7 mL
3	Centrifugation	50 mL	ACD-A	10 mL	60 mL	4 mL
4	Centrifugation	9 mL	Sodium citrate	1 mL	n/a	4–5 mL
5	Filtration	55 mL	ACD-A	5 mL	60 mL	6–8 mL

*The following five commercial systems were prospectively analyzed and labeled as follows: System 1, System 2, System 3, System 4, and System 5. Blood was then obtained from each dog according to the manufacturer’s protocol and specifications for each PRP system. This table summarizes the main characteristics of each system’s protocol*.

## Results

Blood was obtained from a total of 48 different healthy, adult canines. The following breeds were represented: Labrador Retriever (*n* = 7), Border Collie (*n* = 5), German Shepherd Dog (*n* = 4), Golden Retriever (*n* = 4), Rottweiler (*n* = 3), Boxer Dog (*n* = 2), Doberman (*n* = 2), English Bulldog (*n* = 2), Pit Bull Terrier (*n* = 2), American Bulldog (*n* = 1), American Staffordshire Terrier (*n* = 1), Belgian Malinois (*n* = 1), Collie (*n* = 1), French Bulldog (*n* = 1), Great Dane (*n* = 1), Keeshond (*n* = 1), Mastiff (*n* = 1), Newfoundland (*n* = 1), Standard Poodle (*n* = 1), Vizsla (*n* = 1), and mixed canine (*n* = 6). There were 6 intact males, 17 neutered males, 8 intact females, and 17 spayed females. The mean weight was 31.6 kg (range 11.3–55.5 kg). The mean age was 6.2 years old (range 1–13 years old).

### Platelet Concentration

**Figure 1:**
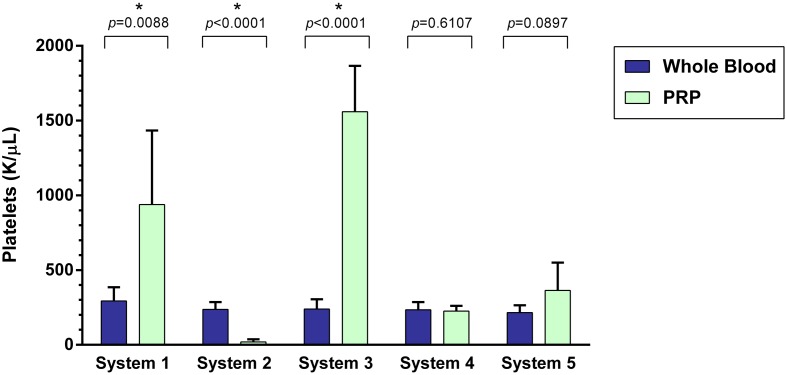
**Comparison of mean whole blood and mean PRP product platelet concentrations between five commercial PRP systems**. An asterisk (*) indicates a statistically significant difference between the mean whole blood and mean PRP product for that PRP system. Bars represent mean ± 95% CI (*n* = 10).

A statistically significant difference was found between the mean whole blood and mean PRP product platelet concentrations of System 1, System 2, and System 3 (Figure 1). Mean platelet concentration was significantly increased for System 1 (*p* = 0.0088), which was a 219% increase, and for System 3 (*p* < 0.0001), a 550% increase. Mean platelet concentration was significantly decreased for System 2 (*p* < 0.0001), which was a 91% decrease. There was no statistically significant change in mean platelet concentration for System 4 (*p* = 0.6107) and System 5 (*p* = 0.0897).

### RBC Concentration

**Figure 2:**
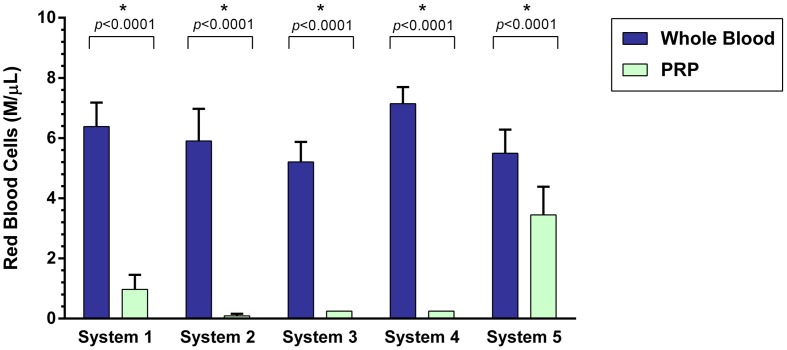
**Comparison of mean whole blood and mean PRP product RBC concentrations between five commercial PRP systems**. An asterisk (*) indicates a statistically significant difference between the mean whole blood and mean PRP product for that PRP system. Bars represent mean ± 95% CI (*n* = 10).

A statistically significant difference was found between the mean whole blood and mean PRP product RBC concentrations of all five PRP systems (Figure 2). Mean RBC concentration was significantly decreased for System 1 (*p* < 0.0001), System 2 (*p* < 0.0001), System 3 (*p* < 0.0001), System 4 (*p* < 0.0001), and System 5 (*p* < 0.0001). System 2 was found to have the greatest decrease in RBC concentration (98%), followed by System 4 (96%), System 3 (95%), System 1 (85%), and System 5 (37%).

### WBC Concentration

**Figure 3:**
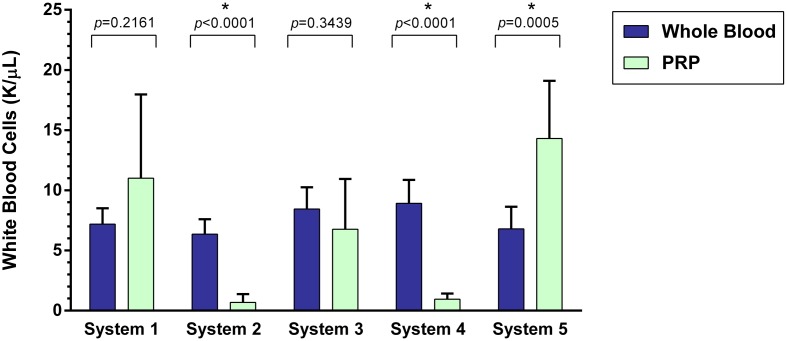
**Comparison of mean whole blood and mean PRP product WBC concentrations between five commercial PRP systems**. An asterisk (*) indicates a statistically significant difference between the mean whole blood and mean PRP product for that PRP system. Bars represent mean ± 95% CI (*n* = 10).

A statistically significant difference was found between the mean whole blood and mean PRP product WBC concentrations of System 2, System 4, and System 5 (Figure 3). Mean WBC concentration was significantly decreased for System 2 (*p* < 0.0001) and System 4 (*p* < 0.0001), an 89% decrease for both systems. Mean WBC concentration was significantly increased for System 5 (*p* = 0.0005), which is a 110% increase. There was no statistically significant change in mean WBC concentration for System 1 (*p* = 0.2161) and System 3 (*p* = 0.3439).

### Neutrophil Concentration

**Figure 4:**
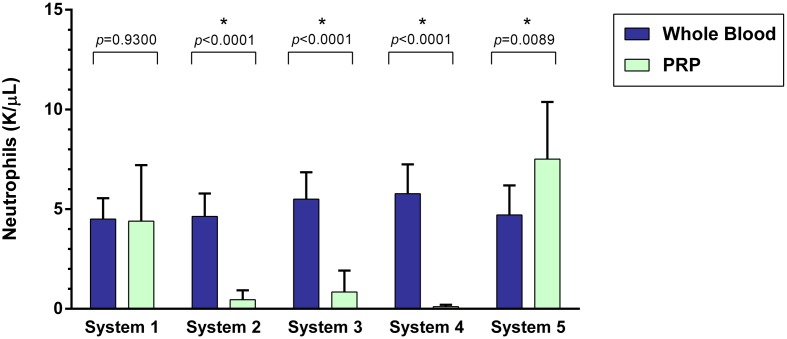
**Comparison of mean whole blood and mean PRP product neutrophil concentrations between five commercial PRP systems**. An asterisk (*) indicates a statistically significant difference between the mean whole blood and mean PRP product for that PRP system. Bars represent mean ± 95% CI (*n* = 10).

A statistically significant difference was found between the mean whole blood and mean PRP product neutrophil concentrations of System 2, System 3, System 4, and System 5 (Figure 4). Mean neutrophil concentration was significantly decreased for System 2 (*p* < 0.0001), System 3 (*p* < 0.0001), and System 4 (*p* < 0.0001). System 2 yielded a 90% decrease, System 3 yielded an 85% decrease, and System 4 yielded a 98% decrease in mean neutrophil concentration compared to mean whole blood values. Mean neutrophil concentration was significantly increased for System 5 (*p* = 0.0089), a 59% increase. There was no statistically significant change in mean neutrophil concentration for System 1 (*p* = 0.9300).

### Monocyte Concentration

**Figure 5:**
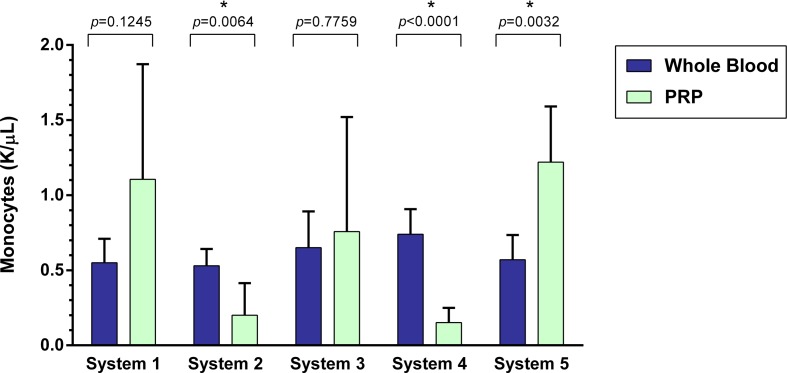
**Comparison of mean whole blood and mean PRP product monocyte concentrations between five commercial PRP systems**. An asterisk (*) indicates a statistically significant difference between the mean whole blood and mean PRP product for that PRP system. Bars represent mean ± 95% CI (*n* = 10).

A statistically significant difference was found between the mean whole blood and mean PRP product monocyte concentrations of System 2, System 4, and System 5 (Figure 5). Mean monocyte concentration was significantly decreased for System 2 (*p* = 0.0064), which was a 62% decrease, and for System 4 (*p* < 0.0001), a 79% decrease. Mean monocyte concentration was significantly increased for System 5 (*p* = 0.0032), a 114% increase. There was no statistically significant change in mean neutrophil concentration for System 1 (*p* = 0.1245) and System 3 (*p* = 0.7759).

### Lymphocyte Concentration

**Figure 6:**
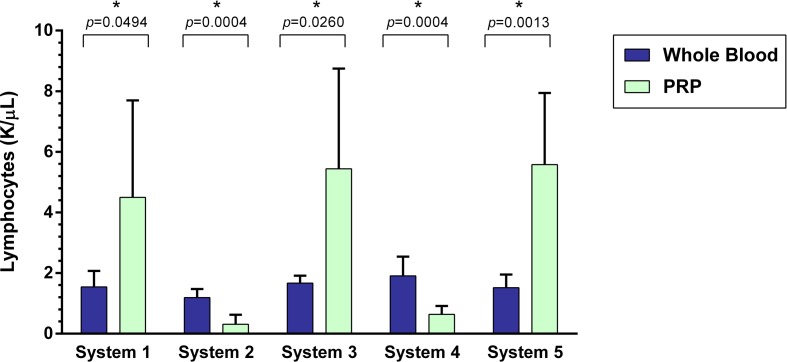
**Comparison of mean whole blood and mean PRP product lymphocyte concentrations between five commercial PRP systems**. An asterisk (*) indicates a statistically significant difference between the mean whole blood and mean PRP product for that PRP system. Bars represent mean ± 95% CI (*n* = 10).

A statistically significant difference was found between the mean whole blood and mean PRP product lymphocyte concentrations of all five PRP systems (Figure 6). Mean lymphocyte concentration was significantly decreased for System 2 (*p* = 0.0004), which is a 74% decrease, and System 4 (*p* = 0.0004), a 67% decrease. Mean lymphocyte concentration was significantly increased for System 1 (*p* = 0.0494), System 3 (*p* = 0.0260), and System 5 (*p* = 0.0013). System 1 yielded a 191% increase, System 3 a 220% increase, and System 5 a 267% increase in mean lymphocyte concentration compared to mean whole blood values.

## Discussion

Platelet-rich plasma concentration results varied among systems. The systems with the highest platelet yield were System 1 and System 3. However, while System 1 yielded a 219% mean increase in platelets and 85% decrease in RBC from baseline, this system failed to reduce neutrophil concentrations. System 3 yields a 550% mean increase in platelets while removing >95% of the RBC and 85% of neutrophils.

Multiple formulations of PRP have been developed and studied. Previous studies in humans have reported that the ideal PRP product should have anywhere from a four- to sevenfold increase in platelets (2–4, 6). However, platelet concentration is not the only important component of a PRP product. Inclusion or exclusion of mononuclear cells, neutrophils, and red blood cells not only define an autologous platelet product, but have also been reported to affect the clinical efficacy of the product and play major roles affecting the inflammatory responses after PRP injection (2, 5, 10, 19–22, 40–44). In this study, the systems with the highest platelet yield were System 1 and System 3.

Reducing RBC concentration is thought to be important when developing the ideal PRP product (40). A recent study revealed an increased RBC concentration in PRP increases the concentrations of unwanted inflammatory mediators, specifically IL-1 and TGF-α. This study also showed that synoviocytes treated with RBC concentrate demonstrated significantly more synoviocyte death when compared with leukocyte-rich PRP (LR-PRP), leukocyte-poor PRP (LP-PRP), and phosphate-buffered saline (PBS) (40). In this study, all five canine PRP systems significantly decreased the RBC concentration.

The effect of leukocyte concentration in PRP products has also been investigated. Both LR-PRP and LP-PRP have been studied and compared. Recent studies have shown LR-PRP is associated with increased pro-inflammatory mediators, including IL-1β, IL-6, IL-8 IFN-γ, and TNF-α (1, 2, 5, 10, 40, 41). Increased leukocytes in PRP are also associated with more metalloproteinase (MMP-3 and MMP-13) gene expression and less cartilage oligomeric matrix protein (COMP) and decorin gene expression (10, 36, 45). These potentially deleterious effects are largely attributed to the presence of neutrophils. Additionally, an increased concentration of neutrophils in PRP is also positively correlated with an increased MMP-9 concentration which degrades collagen and other extracellular matrix molecules (20, 36, 41, 45). One recent study has shown LR-PRP causes significantly more synoviocyte death when compared with LP-PRP and PBS (40). Thus, LP-PRP has been thought to be more beneficial than LR-PRP in maintaining tendon homeostasis and counteract inflammation associated with osteoarthritis (10, 19, 20, 40, 45). Of the systems with the highest platelet yield (System 1 and System 3), System 1 failed to reduce WBC concentration and neutrophil concentrations, while System 3 removed greater than 19% of WBC and 85% of neutrophils.

While an increased neutrophil concentration in PRP is known to have negative effects, the effect of monocytes and lymphocytes remains largely unknown. There was great variation in this study of monocyte and lymphocyte concentrations in the final PRP product. Recent studies suggest that monocytes are associated with an increase in cellular metabolism and collagen production in fibroblasts and a decrease in release of anti-angiogenic cytokines interferon-γ and IL-12 (47, 48). Platelets have also been shown to activate peripheral blood mononuclear cells (lymphocytes, monocytes, and macrophages) to help stimulate collagen production, which is believed to be mediated by an increase in IL-6 expression (47, 48). However, the role of monocytes and lymphocytes in PRP therapy remains unclear and further investigation is warranted.

When compared to the previous study evaluating platelet concentration of PRP products from various canine commercial systems, our study found similar results (49). Three of the commercial systems were tested in both studies. PRP product platelet concentrations and total WBC concentrations were similar between both studies. However, the previous study failed to perform WBC differentiation on the PRP products, so no comparisons can be made regarding neutrophil, lymphocyte, or monocyte concentrations.

Limitations of this study include small sample size and limitations inherent to its design. Different dogs were used for each PRP system, and while all dogs were confirmed to be healthy, one cannot assume that the dogs used are representative of the entire canine population. Ideally, the same dog would be used for all systems and all blood samples would be drawn at the same time to eliminate variation in hematological composition. We attribute the variation in results to this as well as inherent patient factors. However, it was not possible to use the same dogs for simultaneous sample collection and processing as the study was conducted at two separate centers. Furthermore, it was also not feasible to obtain the large blood sample required for all systems to be tested simultaneously.

The goal of this study was to compare key parameters of the PRP product from five commercial canine PRP systems in healthy, adult canines. No claims regarding the efficacy of PRP therapy in canines or the efficacy of the PRP formulations evaluated can be deduced from this study. Further study is indicated to assess the concentrations of growth factors and cytokines in the commercial canine PRP products and to determine the concentration of platelets and growth factors required for therapeutic effect. Further study is also needed to evaluate the efficacy of PRP therapy and further define its clinical applications in canines.

## Author Contributions

All authors assisted with study development, procedures, data analysis, and manuscript preparation.

## Conflict of Interest Statement

Regenerative medicine products and systems were received from the companies below for system validation and clinical testing: Arthrex, EmCyte, Harvest, PulseVet, and Canine Regenerative Therapies.

## Footnotes

^1^SmartPReP^®^2 ACP+, Harvest Technologies, Corp, Plymouth, MA, USA.

^2^Arthrex ACP, Arthrex Orthobiologics, Naples, FL, USA.

^3^CRT Pure PRP, Canine Regenerative Therapies, Fort Meyers, FL, USA.

^4^ProTec PRP, Pulse Veterinary Technologies, LLC, Alpharetta, GA, USA.

^5^C-PET Canine Platelet Enhancement Therapy, Pall Corporation, Port Washington, NY, USA.

^7^Veterinary Orthopedic and Sports Medicine Group, Annapolis Junction, MD, USA.

^8^Idexx LaserCyte Hematology Analyzer, Idexx Laboratories, Westbrook, ME, USA.

^9^GraphPad Prism 6, GraphPad Software Inc, La Jolla, CA, USA.

## Abbreviations

ACP, autologous conditioned plasma; LP-PRP, leukocyte-poor platelet-rich plasma; LR-PRP, leukocyte-rich platelet-rich plasma; PRP, platelet-rich plasma; RBC, red blood cell; WBC, white blood cell.
